# Trends in research on dietary behavior and cardiovascular disease from 2002 to 2022: a bibliometric analysis

**DOI:** 10.3389/fnut.2023.1147994

**Published:** 2023-06-05

**Authors:** Jie Wang, Qingsong Yu, Nannan Liu, Kaidi Nie, Xiaofang Sun, Lina Xia

**Affiliations:** School of Health Preservation and Rehabilitation, Chengdu University of Traditional Chinese Medicine, Chengdu, China

**Keywords:** dietary behavior, cardiovascular disease, bibliometric analysis, VOSviewer, visualization

## Abstract

**Background:**

Dietary behaviors and cardiovascular disease are two major health issues that have attracted a lot of attention from researchers worldwide. In this study, we aimed to provide a comprehensive analysis of the publication trends, authorship patterns, institutional affiliations, country/region contributions, journal outlets, highly cited documents, and keyword clusters in the field of dietary behaviors and cardiovascular disease research over the past two decades.

**Methods:**

We conducted a systematic literature review of peer-reviewed articles published from 2002 to 2022 in the Web of Science Core Collection database. We extracted and analyzed data on the annual publication volume, authorship patterns, institutional affiliations, country/region contributions, journal outlets, highly cited documents, and keyword clusters using bibliometric methods and visualization tools.

**Results:**

Our study analyzed 3,904 articles, including 702 reviews and 3,202 research articles. The results revealed a continuous increase in the number of publications in this field over the past two decades. The top 10 authors, institutions, and countries/regions with the highest publication output were identified, indicating the leading contributors to this field. Moreover, the most frequently cited documents and highly clustered keywords were identified, providing insights into the research themes and topics in this field.

**Conclusion:**

Our study provides a comprehensive analysis of the publication trends, authorship patterns, institutional affiliations, country/region contributions, journal outlets, highly cited documents, and keyword clusters in the field of dietary behaviors and cardiovascular disease research over the past two decades. The findings provide valuable information for researchers, policymakers, and stakeholders to understand the research landscape, identify research gaps, and develop future research directions in this field.

## Introduction

Cardiovascular diseases (CVDs) continue to pose a significant global health burden, contributing to substantial morbidity and mortality worldwide (1). The prevalence of CVDs has reached epidemic proportions, warranting urgent attention and comprehensive strategies for prevention and management (2). Among the various modifiable risk factors associated with CVDs, dietary behaviors have emerged as crucial contributors to the development and progression of these conditions (3).

A growing body of evidence suggests that an individual’s dietary choices and patterns play a pivotal role in determining their cardiovascular health status (4). The intricate interplay between nutrients, bioactive compounds, and dietary components can significantly influence the pathophysiological processes underlying CVDs, including inflammation, oxidative stress, endothelial dysfunction, dyslipidemia, and hypertension (5–8). Furthermore, the impact of dietary factors extends beyond traditional risk factors, encompassing novel markers such as gut microbiota composition, metabolomic profiles, and epigenetic modifications, which further shape an individual’s cardiovascular risk profile (9–11).

Understanding the intricate relationship between dietary behaviors and cardiovascular health is essential for the development of effective preventive and therapeutic interventions (12). Moreover, unraveling the mechanisms underlying these associations can provide valuable insights into the etiology of CVDs and guide personalized dietary recommendations tailored to individuals at risk (13, 14). Additionally, exploring the cultural and social determinants that shape dietary behaviors is crucial for addressing health disparities and promoting equitable access to cardiovascular health (15).

Thus, this study aims to conduct a bibliometric analysis of studies examining dietary behavior and cardiovascular disease, published from 2002 to 2022, utilizing the Web of Science database. This study examines information on keywords, authors, institutions, countries/regions, and journals, employing VOSviewer to analyze the data. Ultimately, a deeper understanding of the intricate relationship between dietary behaviors and cardiovascular health can inform evidence-based strategies to promote heart health, empower individuals to make informed dietary choices, and alleviate the burden of cardiovascular diseases on a global scale.

## Materials and methods

### Data source

**Figure 1:**
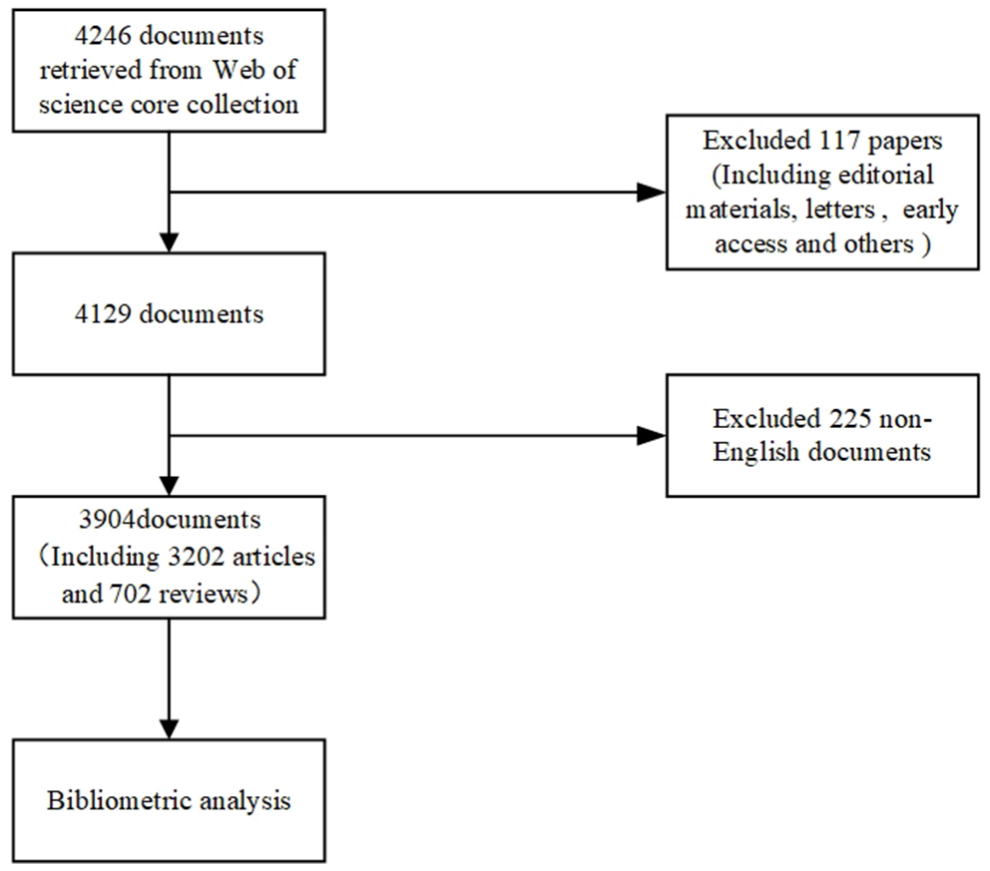
Research flowchart.

The research process is illustrated in Figure 1. We collected the data from the Web of Science Core Collection database. The timespan covered the last 21 years (from 2002 to 2022). The “topic” field was used to search for articles related to a specific research field. TS = (“behavior, feeding” or “feeding behaviors” or “eating behavior” or “behavior, eating” or “eating behaviors” or “feeding-related behavior” or “behavior, feeding-related” or “feeding related behavior” or “feeding-related behaviors” or “feeding patterns” or “feeding pattern” or “pattern, feeding” or “food habits” or “food habit” or “habit, food” or “eating habits” or “eating habit” or “habit, eating” or “dietary habits” or “dietary habit” or “habit, dietary” or “diet habits” or “diet habit” or “habit, diet” or “habits, diet”) and (“cardiovascular disease” or “disease, cardiovascular” or “cardiovascular diseases” or “diseases, cardiovascular”). The “document type” field was set to “article” and “review” to ensure that we only included articles in our analysis. The language of the included literature was limited to English in order to ensure consistency and minimize potential language bias.

### Data analysis

We used VOSviewer, a bibliometric analysis software, to analyze the data. VOSviewer uses co-occurrence analysis to identify relationships between different terms and keywords in the articles. The software generates a map that shows clusters of related terms. The size of each term on the map indicates its importance and the thickness of the lines between the terms represents the strength of the relationship between them.

To generate the map, we first exported the search results from Web of Science Core Collection into a text file and then imported it into VOSviewer. We conducted keyword clustering analysis using the following parameters, with a minimum occurrence frequency set at 50 for each term to be included in the analysis. We used the VOSviewer default settings for the remaining parameters.

### Data visualization

We used the VOSviewer software to generate the visualization of the co-occurrence analysis results. The visualization is presented in a map format, where each cluster of keywords is represented by a different color. The size of each keyword on the map is proportional to its frequency of occurrence in the articles, and the proximity of two keywords on the map indicates their degree of co-occurrence.

## Results

### Annual publication volume

**Figure 2:**
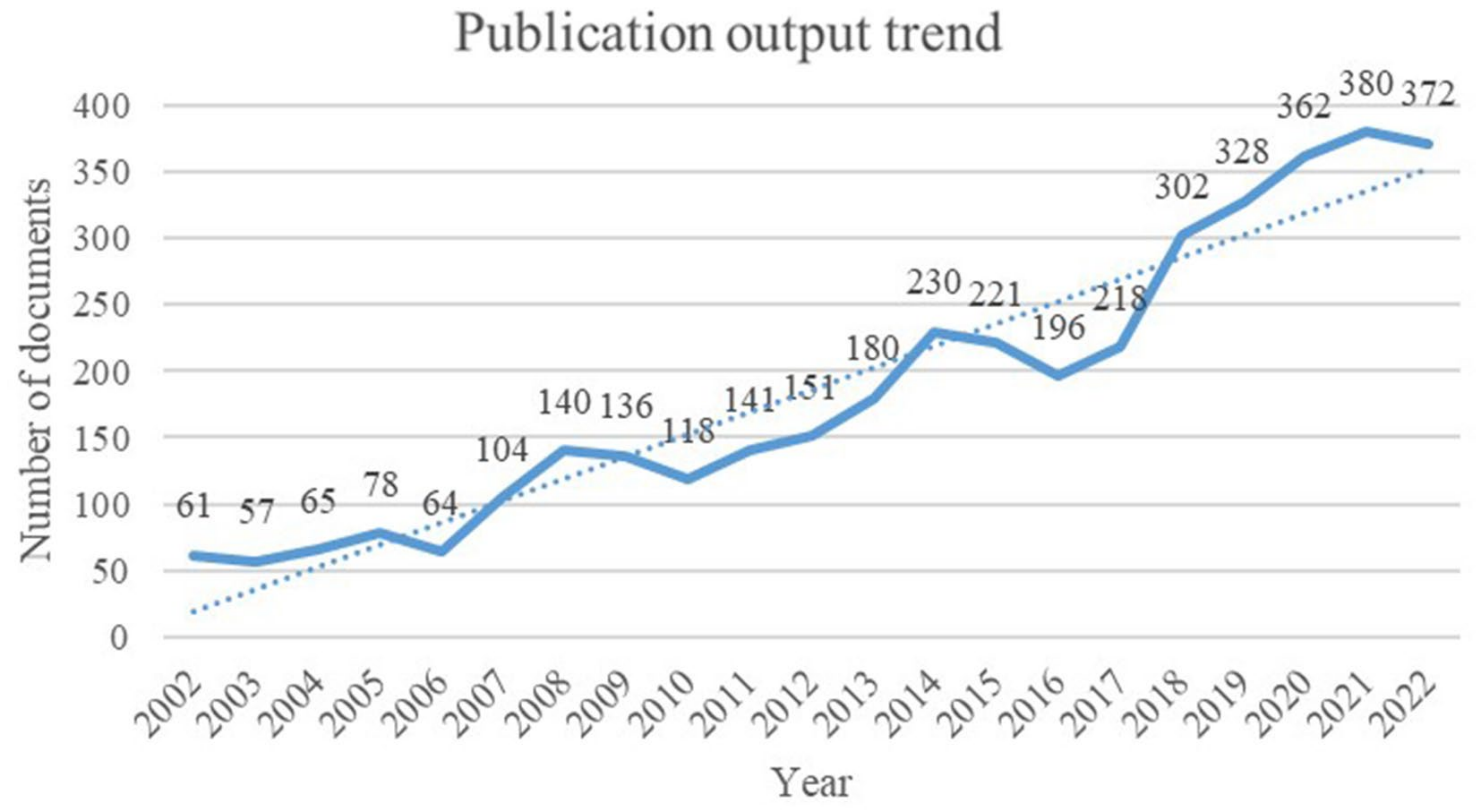
Trend of publications on dietary behavior and cardiovascular disease from 2002 to 2022.

In our study, we analyzed a total of 3,904 articles consisting of 702 reviews and 3,202 articles within the field of dietary behaviors and cardiovascular diseases. Our findings reveal that the number of publications in this area has been on the rise from 2002 to 2022. This trend is visually presented in Figure 2, which illustrates the number of publications related to dietary behaviors and cardiovascular research from 2002 to 2022.

### Author analysis

**Figure 3:**
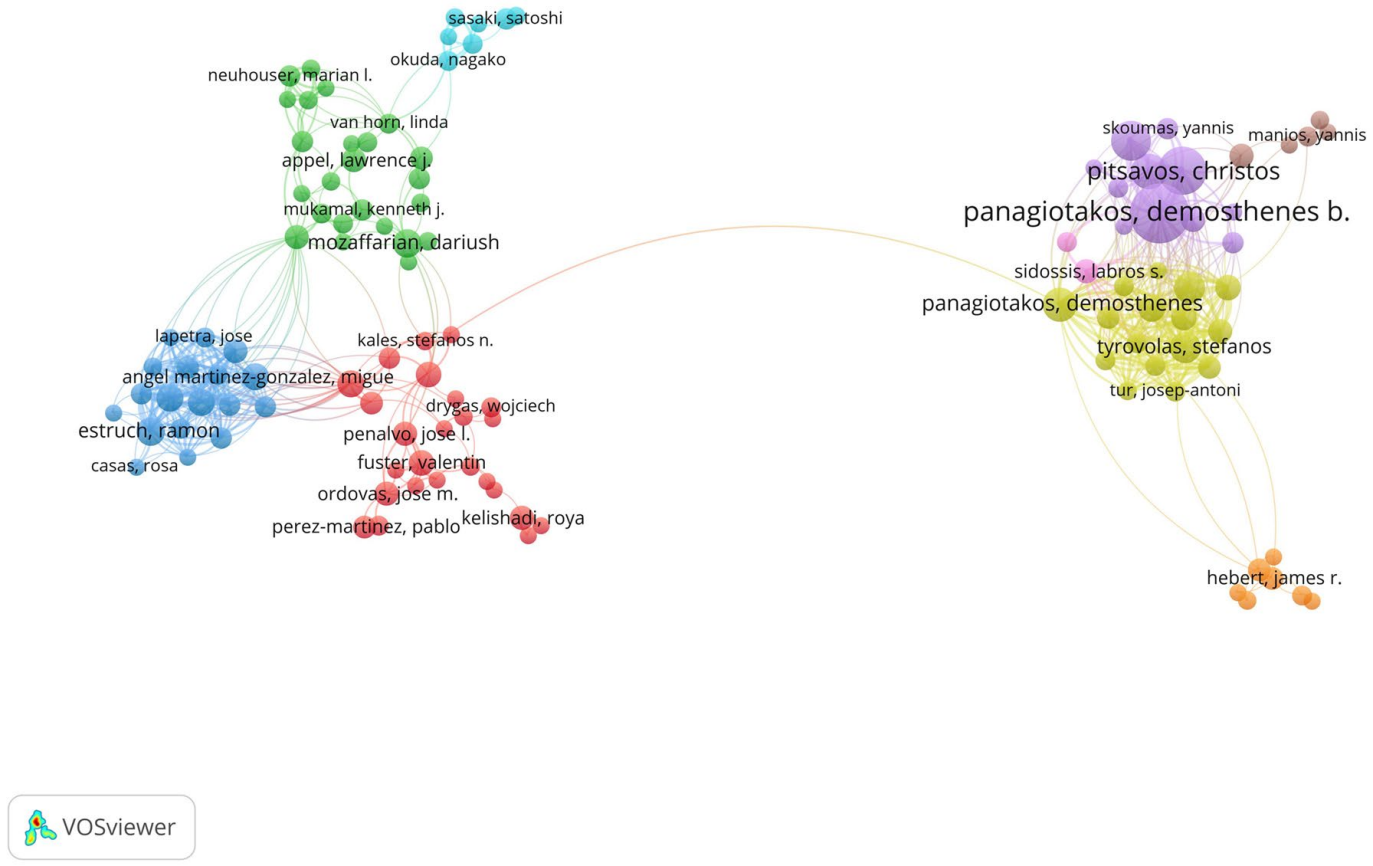
The co-authorship of authors.

From 2002 to 2022, a total of 20,640 authors have published documents in this field. Table 1 presents the top 10 authors ranked by the number of publications in the field of dietary behavior and cardiovascular disease. At the top of the list is Panagiotakos DB, who has published a remarkable 83 documents, indicating his significant contribution to the field. Pitsavos C follows closely behind with 57 publications, while Stefanadis C ranks third with 47 publications. Figure 3 shows the co-authorship of the authors.

**Table 1:** Top 10 authors ranked by the number of publications.

Ranking	Authors	Documents
1	Panagiotakos DB	83
2	Pitsavos C	57
3	Stefanadis C	47
4	Chrysohoou C	40
5	Martinez-Gonzalez MA	34
6	Panagiotakos DB	24
7	Iacoviello L	20
8	Polychronopoulos E	19
9	Tyrovolas S	19
10	Zeimbekis A	18

### Institutional analysis

**Figure 4:**
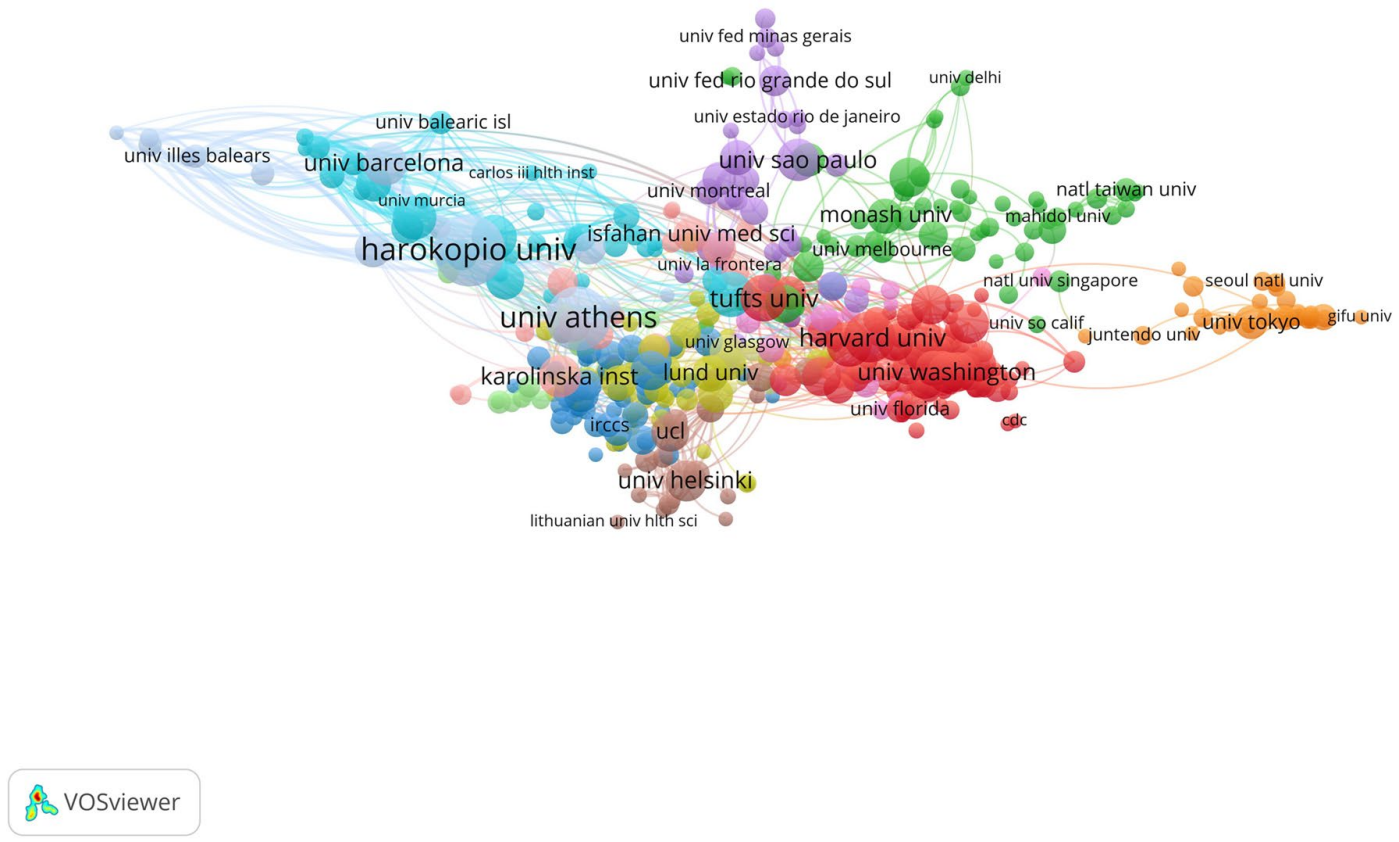
The co-authorsship map of institutions.

In the past 21 years, a total of 5,244 research institutions have published papers in this field. Table 2 presents the top 10 institutions with the highest number of publications in the field of study. Harvard University leads the list with a remarkable 147 publications, followed closely by Harokopio University Athens with 124 publications. Ciber Centro de Investigacion Biomedica En Red ranks third with 116 publications, while National Kapodistrian University of Athens and University of California System round out the top five with 111 and 97 publications, respectively. The remaining institutions in the list include Athens Medical School, University of London, Harvard T. H. Chan School of Public Health, Harvard Medical School, and Ciberobn, all of which have made significant contributions to the field through their numerous publications. Figure 4 shows the co-authorship of institutions.

**Table 2:** Top 10 institutions with the highest number of publications.

Ranking	Institution	Documents
1	Harvard University	147
2	Harokopio University Athens	124
3	Ciber Centro de Investigacion Biomedica En Red	116
4	National Kapodistrian University of Athens	111
5	University of California System	97
6	Athens Medical School	88
7	University of London	86
8	Harvard T. H. Chan School of Public Health	85
9	Harvard Medical School	80
10	Ciberobn	74

### Journal analysis

**Figure 5:**
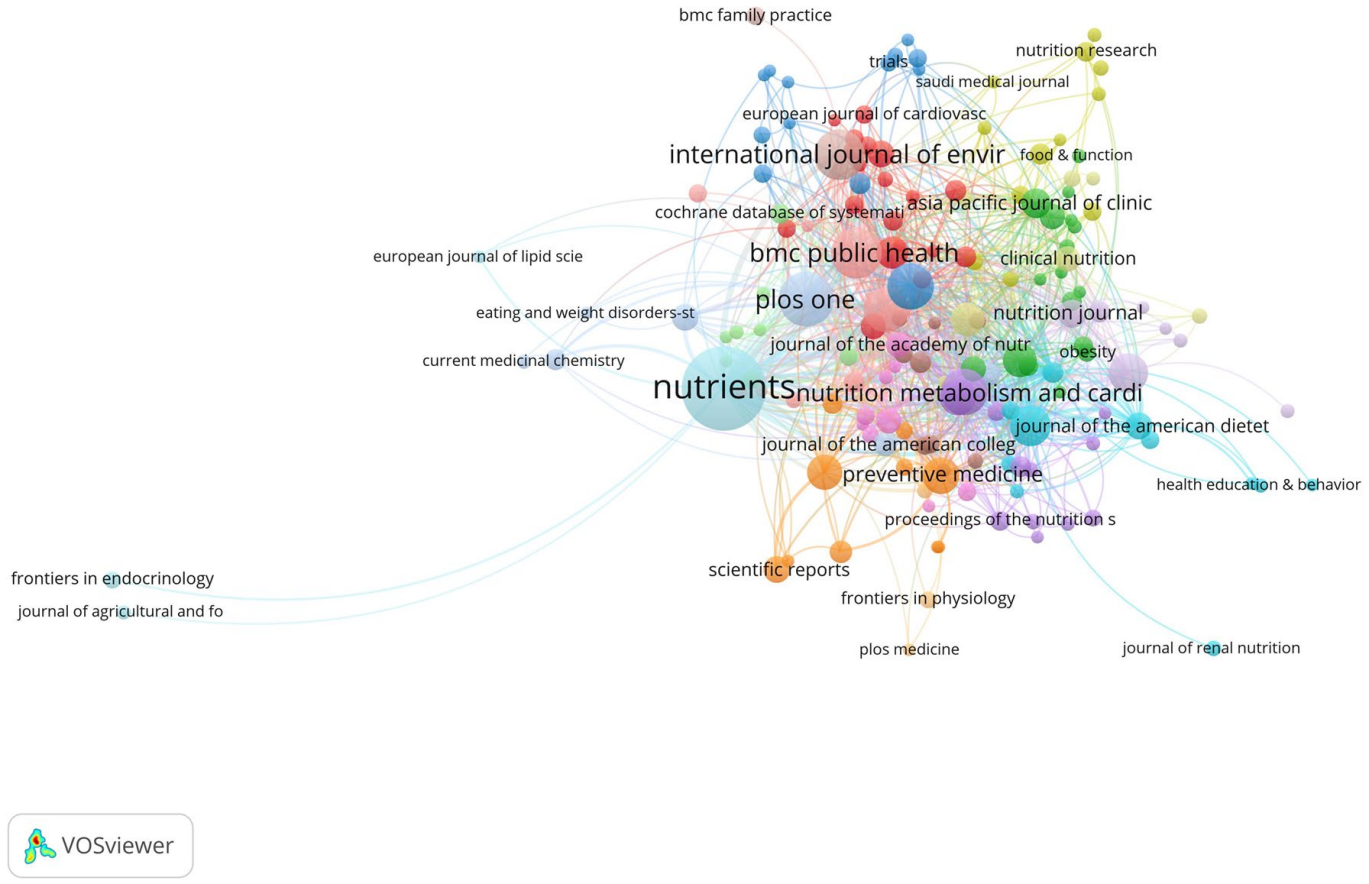
Visualization map of journal citations.

A total of 1,301 journals have published papers related to the field. Table 3 presents the top 10 journals ranked by the number of publications in this field. The journal with the highest number of publications is Nutrients, with 192 papers. Following closely is PLoS One with 80 publications, and BMC Public Health and International Journal of Environmental Research and Public Health with 70 papers each. Other journals that made the top 10 list include Public Health Nutrition, Nutrition Metabolism and Cardiovascular Diseases, European Journal of Clinical Nutrition, American Journal of Clinical Nutrition, and Journal of Nutrition. The Figure 5 shows the visualization map of journal citations.

**Table 3:** Top 10 journals ranked by number of publications.

Ranking	Journal	Documents
1	Nutrients	192
2	PLoS One	80
3	BMC Public Health	70
4	International Journal of Environmental Research and Public Health	70
5	Public Health Nutrition	60
6	Nutrition Metabolism and Cardiovascular Diseases	58
7	European Journal of Clinical Nutrition	56
8	American Journal of Clinical Nutrition	45
9	Journal of Nutrition	42
10	Nutrients	37

### Country/regions analysis

**Figure 6:**
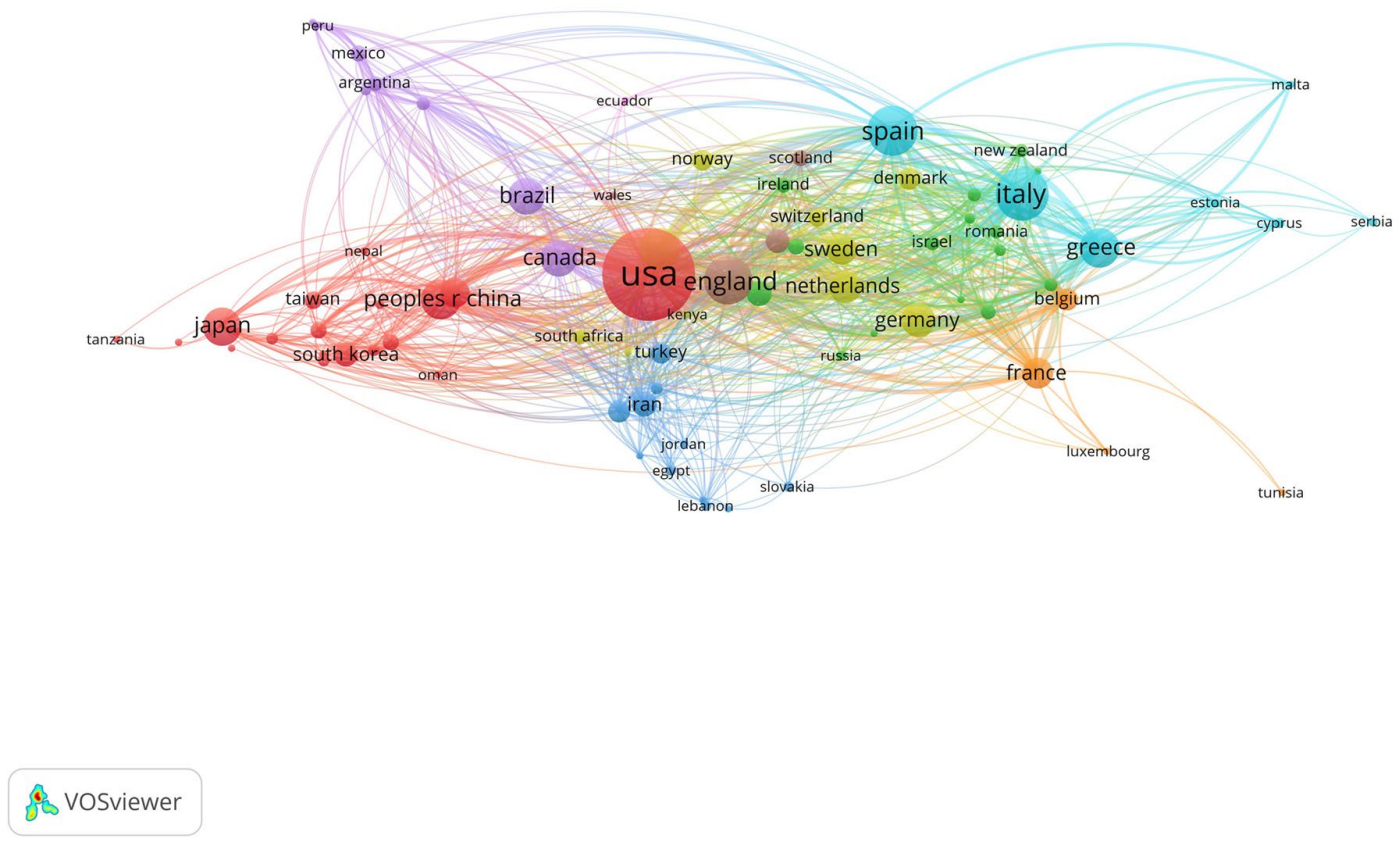
The co-author ship map of countries/regions.

In the past 21 years, a total of 121 countries/regions have published papers related to this field. Table 4 presents the top 10 countries/regions ranked by the number of publications in this field. The United States holds the leading position with 1,102 publications, followed by Italy with 371 publications and Spain with 325 publications. England, Australia, and People’s Republic of China also have a significant number of publications with 302, 221, and 210 documents, respectively. Greece, Japan, Canada, and Brazil are also among the top 10 countries/regions. Figure 6 shows the co-authorship of countries/regions.

**Table 4:** Top 10 countries/regions by number of publications.

Ranking	Country/region	Documents
1	United States	1,102
2	Italy	371
3	Spain	325
4	England	302
5	Australia	221
6	People’s Republic of China	210
7	Greece	204
8	Japan	186
9	Canada	180
10	Brazil	172

### Documents analysis

**Figure 7:**
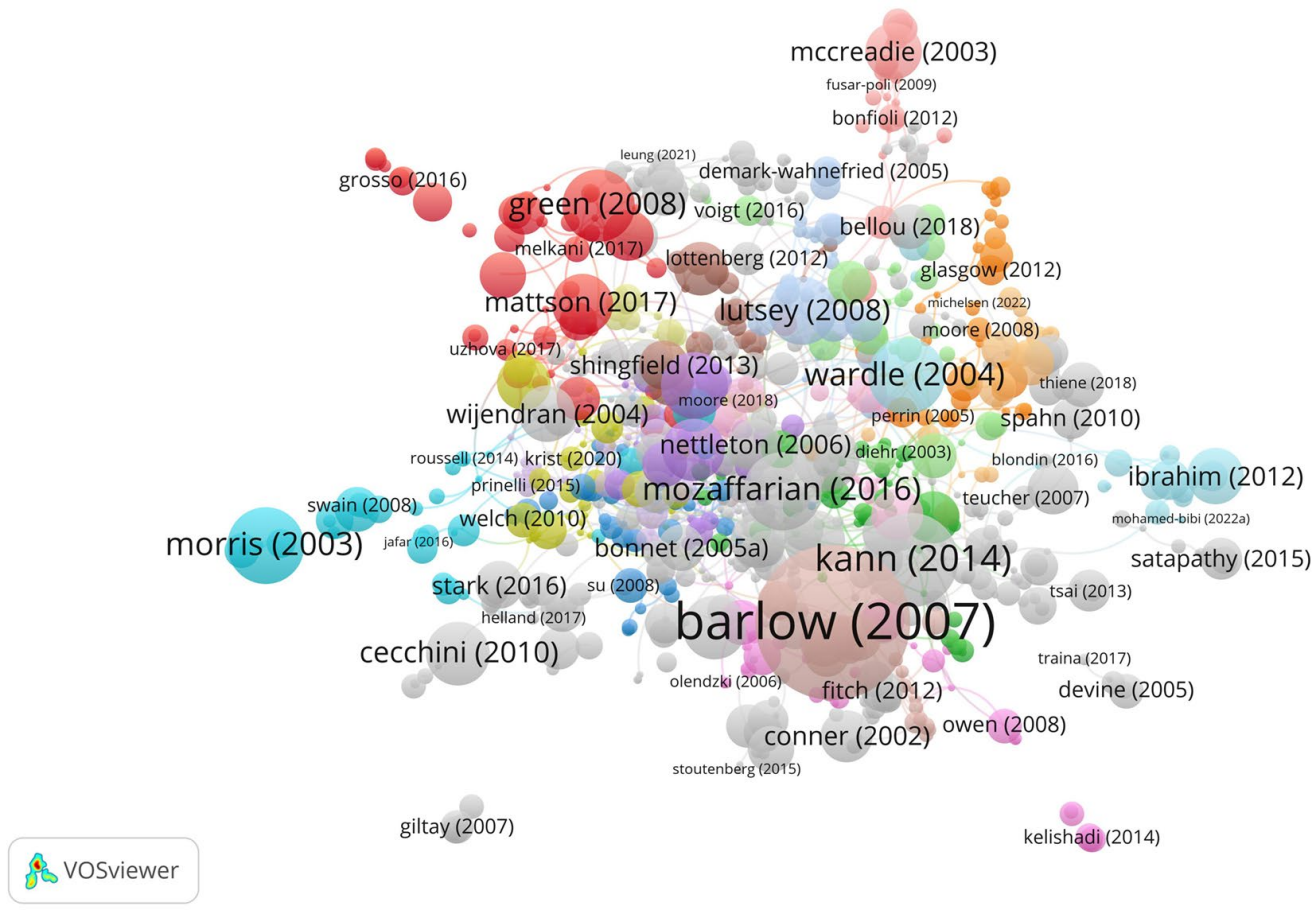
Visualization knowledge maps of highly cited documents.

In the past 21 years, a total of 3,904 papers related to this field have been published. Table 5 shows the top 10 highly cited documents in the field. The expert committee recommendations regarding the prevention, assessment, and treatment of child and adolescent overweight and obesity: summary report, published in Pediatrics in 2007, ranked first with 3,119 citations. The report provides comprehensive recommendations on the prevention, assessment, and treatment of child and adolescent obesity, which is a major public health problem worldwide. The second most cited document is Youth Risk Behavior Surveillance—United States, 2015, published in MMWR Surveillance Summaries in 2016, with 1,223 citations. The document presents the results of a survey of risk behaviors among high school students in the United States, including behaviors related to obesity, physical activity, and nutrition. Ranked third is Youth Risk Behavior Surveillance—United States, 2013, published in Sports Medicine in 2014, with 1,143 citations. Similar to the second-ranked document, it reports on the results of a survey of risk behaviors among high school students in the United States. Figure 7 shows the visualization knowledge maps of highly cited documents.

**Table 5:** Top 10 highly cited documents.

Ranking	Title	Journal	Citation	Year
1	Expert committee recommendations regarding the prevention, assessment, and treatment of child and adolescent overweight and obesity: summary report	Pediatrics	3,119	2007
2	Youth risk behavior surveillance—United States, 2015	MMWR Surveillance Summaries	1,223	2016
3	Youth risk behavior surveillance—United States, 2013	Sports Medicine	1,143	2014
4	Central effects of stress hormones in health and disease: understanding the protective and damaging effects of stress and stress mediators	European Journal of Pharmacology	1,134	2008
5	Diabetic nephropathy: diagnosis, prevention, and treatment	Diabetes Care	1,111	2005
6	Dietary and policy priorities for cardiovascular disease, diabetes, and obesity a comprehensive review	Circulation	809	2016
7	Gender differences in food choice: the contribution of health beliefs and dieting	Annals of Behavioral Medicine	771	2004
8	Consumption of fish and n-3 fatty acids and risk of incident Alzheimer disease	Archives of Neurology	765	2003
9	The meter of metabolism	Cell	683	2008
10	Dietary intake and the development of the metabolic syndrome—the atherosclerosis risk in communities study	Circulation	609	2008

### Keyword analysis

**Figure 8:**
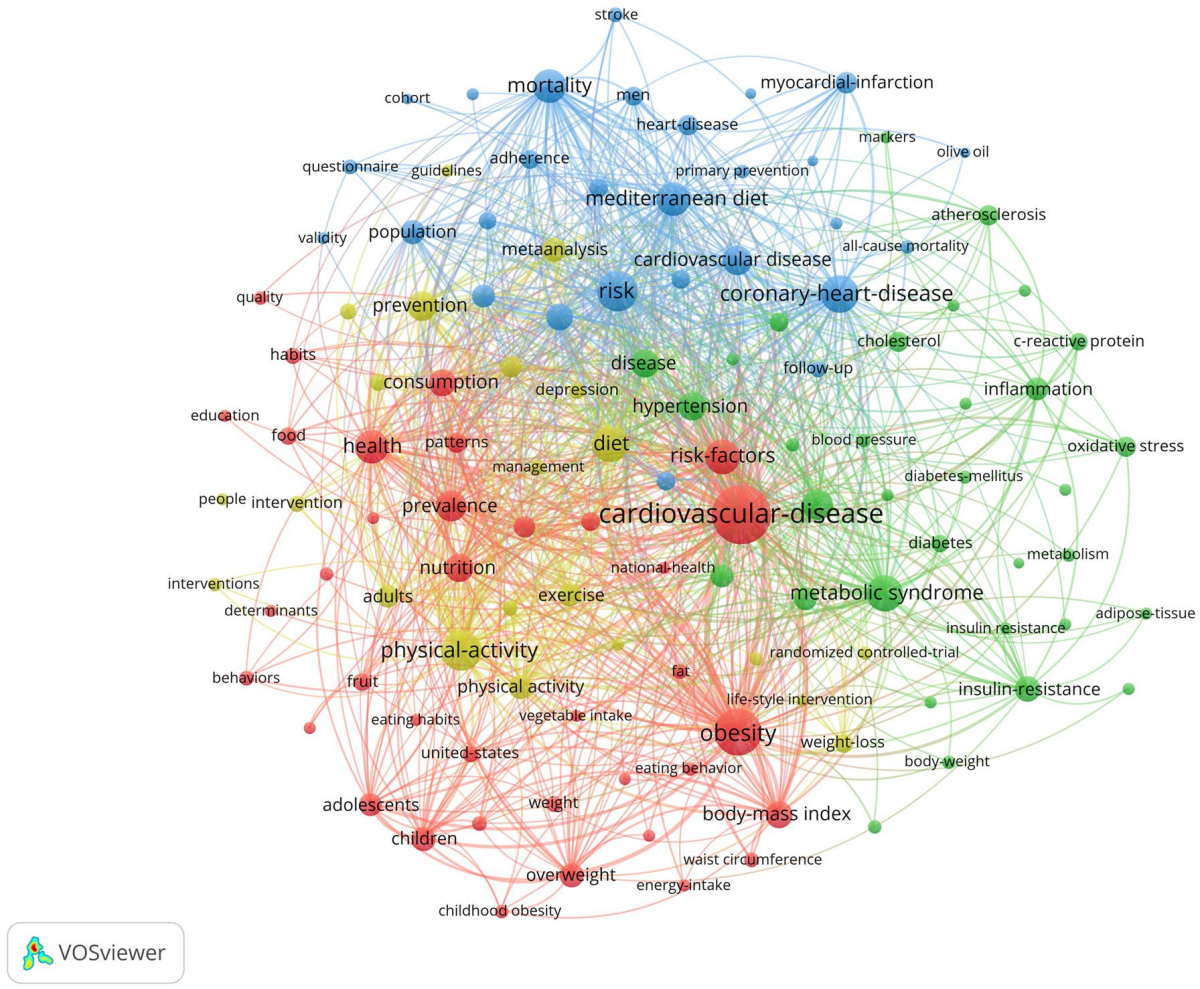
Visualization of co-occurrence analysis for keywords.

There are a total of 12,855 keywords. Figure 8 illustrates the results of the keyword clustering analysis, where the keywords are categorized into four clusters represented by the colors red, blue, yellow, and green. The red cluster is dominated by keywords such as cardiovascular disease, obesity, body mass index, and overweight. The blue cluster includes keywords such as Mediterranean diet, heart disease, olive oil, mitochondrial dysfunction, stroke, and coronary heart disease. The yellow cluster is mainly composed of keywords such as diet, exercise, intervention, and prevention. The green cluster includes keywords such as oxidative stress, metabolic syndrome, insulin resistance, adipose tissue, blood pressure, cholesterol, inflammation, and C-reactive protein. This analysis provides a comprehensive understanding of the key themes and concepts in the field of diet and cardiovascular disease research.

## Discussion

### General information

This study analyzed the literature related to diet behavior and cardiovascular disease over the past 21 years, revealing a trend of increasing research output in this field. This suggests a growing interest among researchers and the general public in the link between diet behavior and cardiovascular health. Notably, Panagiotakos DB emerged as one of the most prolific authors in this field, having published 83 articles that highlight his significant contributions. Collaboration among authors also emerged as an important factor in advancing research in this area. Harvard University, Harokopio University Athens, and Ciber Centro de Investigacion Biomedica En Red were the top institutions in terms of research output, indicating their pivotal role in this field. Meanwhile, contributions from diverse institutions worldwide underscore the global reach of this research. The United States led the way in research output with 1,102 published articles, followed by Italy, Spain, England, Australia, and mainland China. This highlights the global nature of research in this field and the widespread interest among researchers worldwide. Nutrients, PLoS One, BMC Public Health, and International Journal of Environmental Research and Public Health were among the most prolific journals publishing in this field, covering a wide range of topics related to diet behavior and cardiovascular health. Keyword analysis revealed 12,855 keywords clustered into four groups represented by red, blue, yellow, and green. Red keywords included those related to cardiovascular disease, obesity, body mass index, and overweight. Blue keywords included Mediterranean diet, heart disease, olive oil, mitochondrial dysfunction, stroke, and coronary heart disease. Yellow keywords were related to diet, exercise, intervention, and prevention. Green keywords included oxidative stress, metabolic syndrome, insulin resistance, adipose tissue, blood pressure, cholesterol, inflammation, and C-reactive protein.

### Research trends, knowledge gaps, and future directions

Taken together, these findings highlight the expanding scope of research on diet behavior and cardiovascular disease, encompassing a wide range of topics and concepts. Notable contributions from authors, institutions, and countries, such as Panagiotakos DB, Harvard University, and the United States, provide valuable insights for future research and practice to reduce the incidence of cardiovascular disease and promote public health.

Although the relationship between diet behavior and cardiovascular disease has been extensively studied, there are still some gaps that need to be further explored. One of these gaps is the impact of diet on the risk of cardiovascular disease in different races and cultural backgrounds. While some studies have suggested a correlation between dietary habits and the incidence of cardiovascular disease, most of these studies have focused on Western populations (16–18), and further research is needed for other ethnic and cultural groups. Another gap is the personalized design of dietary intervention measures. While some dietary intervention measures have been shown to improve the risk of cardiovascular disease (19–21), there is significant biological variation between individuals. Therefore, further research is needed to explore how to design personalized dietary intervention measures based on individual characteristics such as genes, metabolites, and microbiomes. Based on the trends and gaps in research, we can predict future research directions and focuses in this field. Firstly, we can expect to see more research on the relationship between dietary behavior and cardiovascular health in different cultural and geographical contexts. Another possible direction is the study of differences in dietary behavior and cardiovascular health between different individuals. This includes research on individuals of different genders, ages, ethnicities, and health statuses, as well as differences between dietary habits and nutrient requirements. Overall, we expect the research field of the relationship between dietary behavior and cardiovascular disease to continue to develop and expand, with future research focusing more on individual differences, the relationship between dietary patterns and nutrients, and the effects of food processing and cooking.

### Strengths and limitations

In this study, a comprehensive analysis of publication trends, authorship patterns, institutional affiliations, country/region contributions, journal outlets, highly cited documents, and keyword clusters in the field of dietary behaviors and cardiovascular disease research over the past two decades was conducted. The use of systematic literature review and bibliometric methods ensured a rigorous and objective approach to analyzing the 3,904 articles included in the study, providing a representative sample of the research in this field. However, the limitations of this study include the reliance on articles from the Web of Science Core Collection database, which may not encompass all relevant publications, and the restriction to publications in English, which may limit generalizability to non-English speaking countries.

## Conclusion

In conclusion, our analysis of the literature related to diet behavior and cardiovascular disease over the past 21 years reveals a trend of increasing research output in this field, indicating a growing interest among researchers and the general public. Notable contributions from authors, institutions, and countries provide valuable insights for future research and practice to reduce the incidence of cardiovascular disease and promote public health. However, there are still gaps in our understanding of the relationship between diet behavior and cardiovascular disease that need to be addressed in future research. We expect the research field in this area to continue to develop and expand, with a focus on individual differences, the relationship between dietary patterns and nutrients, and the effects of food processing and cooking.

## Data availability statement

The raw data supporting the conclusions of this article will be made available by the authors, without undue reservation.

## Author contributions

JW served as the first author and wrote the manuscript. QY contributed to the analysis and interpretation of the data and provided valuable insights during the manuscript writing process. NL and KN conducted the literature search and assisted in the drafting of the manuscript. XS provided valuable insights and expertise in data analysis, refining the research methodology. XS also played a crucial role in editing the manuscript to ensure clarity and coherence. LX as the corresponding author, reviewed and revised the manuscript and oversaw the overall process, providing guidance and coordination among the authors. All authors contributed to the article and approved the submitted version.

## Funding

This work was financially supported by National Natural Science Foundation of China (82274384).

## Conflict of interest

The authors declare that the research was conducted in the absence of any commercial or financial relationships that could be construed as a potential conflict of interest.

## Publisher’s note

All claims expressed in this article are solely those of the authors and do not necessarily represent those of their affiliated organizations, or those of the publisher, the editors and the reviewers. Any product that may be evaluated in this article, or claim that may be made by its manufacturer, is not guaranteed or endorsed by the publisher.
